# Enhancing concrete support bearing strength with strain hardening cementitious composite (SHCC) layer: an experimental study

**DOI:** 10.1038/s41598-025-26427-w

**Published:** 2025-12-03

**Authors:** Sabry Fayed, Mohamed Elkafrawy, Mohammed Elsharkawy

**Affiliations:** 1 Civil Engineering Department, Faculty of Engineering, Kafrelsheikh University, Kafrelsheikh, Egypt; 2 Structural Engineering Department, Faculty of Engineering, Tanta University, Tanta, Egypt

**Keywords:** Bearing behavior, Local pressure, Concrete supports, SHCC, Grooves, Engineering, Materials science

## Abstract

This study investigates the potential of Strain Hardening Cementitious Composite (SHCC) as a reinforcement layer to enhance the bearing strength of concrete supports, addressing the brittle failures often observed in conventional concrete under concentrated loads. Specimens were divided into six groups (G1 to G6) to explore variables such as groove number and size, SHCC layer depth and size, and the use of anchors. The results demonstrated that incorporating SHCC layers into concrete blocks effectively reduced crack width and delayed the onset of ultimate failure. However, excessive SHCC thickness compromised stress distribution, leading to earlier and more brittle failure. Specifically, increasing the number and size of grooves enhanced bearing capacity by an average of 16.7% and 22.4%, respectively. Similarly, using appropriate size of SHCC layer improved stress distribution, resulting in a 36.2% increase in bearing capacity. Conversely, increasing SHCC depth at the expense of its size reduced bearing capacity by 29.4%, likely due to inefficient stress distribution. Although the use of anchors was intended to enhance the bond between the SHCC layer and the underlying concrete, they instead acted as stress concentrators, accelerating failure and reducing bearing strength by 17.6% on average. Similarly, while the addition of grooves and optimized SHCC block sizes led to substantial gains in Energy Absorption Capacity (EAC), other modifications, such as anchoring or inappropriate SHCC layer depths, had counterproductive effects. The study concludes with the proposal of an empirical formula to predict the bearing capacity of SHCC-reinforced structures, incorporating the key test parameters identified.

## Introduction

Understanding the bearing strength of concrete is crucial for ensuring the structural integrity and safety of various construction and infrastructure projects, including column-foundation interfaces, corbels, bridge pedestals, support anchorages, and post-tensioned members^1,2^. Bearing strength is particularly vital in scenarios where the load is concentrated on a small area, as the pressure exerted can be significantly high. This strength is determined primarily by the concrete’s compressive strength and the ratio of the total concrete surface area to the loaded steel bearing area^3^.

Critical factors influencing bearing strength include the dimensions of the concrete block specimens and the size of the bearing steel plate^4^. Additionally, the shape of a concrete specimen is a key determinant of its strength indicators, with variations in shape significantly altering the outcomes^5,6^. The confinement effects, varying with the height of concrete blocks, enhance the bearing strength by providing lateral support that helps distribute stresses more evenly throughout the material^7^. To accurately assess concrete’s bearing strength, various testing methods such as compression tests, bearing tests, and pull-out tests are employed^8–17^. These tests provide valuable insights into the structural capabilities and limitations of concrete under different loading conditions.

Extensive experimental studies by researchers like Meyerhof and Shelson have examined various failure modes of concrete under concentrated loads^18,19^. Their observations revealed patterns resembling those seen in triaxial compression tests, highlighting consistent mechanical behaviors under different stress conditions. Simultaneously, Hawkins developed approximate formulas to predict the bearing strength of concrete members when loaded through rigid plates, derived from analyzing the outcomes of 230 tests that varied in loading geometry, specimen size, and both the type and strength of the concrete used^20^. These findings provide a broad empirical basis for understanding concrete behavior under load. Building on these insights, Au and Baird proposed a theoretical model to explain bearing failure in concrete blocks, centered on the concept of an inverted pyramid forming beneath the area of localized load^21^. This model suggests that as the pyramid shape penetrates downward, it induces horizontal pressures resulting in a complex state of bending and tension within the concrete block. The model posits that failure occurs when the maximum tensile stress at the top of the block exceeds the concrete’s tensile strength, providing a nuanced view of the structural dynamics leading up to concrete failure.

Conventional concrete is known for its compressive strength but suffers from significant limitations due to its inherent brittleness and poor tensile strength^22^. These characteristics lead to specific failure mechanisms under bearing loads, notably the splitting of concrete^23^. This splitting typically occurs due to lateral tensile stresses induced by the vertical compression of the material, an effect compounded by concrete’s low ductility. Under load, concrete cannot deform significantly before failure, leading to abrupt and unpredictable cracking and failure. The brittleness index of concrete is calculated based on peak compressive strength and residual compressive strength, and it increases with the decrease of peak strain, energy absorption, and fractal dimension of fracture^24^. In structures where the bearing loads induce significant bending or stretching, these vulnerabilities can be particularly problematic, often necessitating additional reinforcements or the adoption of more advanced materials to ensure structural integrity and safety. Tests on interfaces reinforced with steel bars or industrial steel screws have shown substantial interface resistance, suggesting solutions for thin concrete overlays added to existing members^4^. Reinforcing trapezoidal filling structures with tire slices has also been shown to significantly reduce settlement and improve ultimate bearing capacity^25^.

The emergence of Strain Hardening Cementitious Composites (SHCC) marks a significant advancement in construction materials, offering properties superior to traditional concrete^26^. Characterized by unique microstructural features, SHCC has been extensively studied for its superior ductility and remarkable tensile strength, qualities significantly lacking in traditional concrete^27^. These composites, engineered to include fibers, provide improved crack resistance and allow the material to exhibit strain-hardening behavior under loading conditions. This capacity enables SHCC to develop multiple fine cracks instead of single large cracks, effectively distributing stresses and maintaining structural integrity under mechanical stresses. The extensive research surrounding SHCC underscores its potential to overcome many limitations of conventional concrete, such as brittleness and poor tensile performance^28–35^. As a result, SHCC is increasingly considered for applications where enhanced durability and resilience are critical, making it a focal point for recent studies aiming to push the boundaries of what can be achieved with cementitious construction materials.

This study investigated the effectiveness of employing a SHCC layer as an innovative technique to improve the distribution of concentrated loads on small areas of conventional concrete block supports. The principal aim was to counteract the typical brittle failures associated with conventional concrete, such as cracking and splitting, by leveraging the superior properties of SHCC. Specifically, SHCC’s enhanced ductility and microcracking capabilities allowed it to absorb and redistribute stress more effectively, potentially increasing the overall bearing strength of the concrete structure. In evaluating the efficacy of SHCC layers, the research focused on several critical test parameters: the thickness and dimensions of the SHCC layer, which were hypothesized to play significant roles in the load-bearing capacity and distribution; the grooving conditions between the SHCC layer and the underlying conventional concrete, which may influence the mechanical bond and load transfer efficiency between the two materials; and the anchoring mechanisms employed, which are essential for ensuring that the SHCC layer remains securely attached to the concrete substrate under load. By systematically analyzing these parameters, the study aimed to delineate the conditions under which SHCC could most effectively enhance the durability and strength of conventional concrete supports, thus providing valuable insights for the construction industry in designing more resilient and longer-lasting structures.

## Aim of the study

The primary objective of this study was to explore the innovative use of SHCC as a reinforcement layer in conventional concrete block supports. This research focused on how an SHCC layer could enhance the distribution of concentrated loads and mitigate common brittle failures such as cracking and splitting, which are prevalent in conventional concrete structures. By leveraging the exceptional ductility and ability of SHCC to develop multiple fine cracks, the study aimed to increase the overall bearing strength and extend the lifespan of concrete supports. The novelty of this research lies in its application of SHCC in a layered approach; an underexplored method for improving load distribution and structural integrity under concentrated stresses. Despite the extensive research on SHCC’s material properties and performance under general loading conditions, a considerable knowledge gap existed regarding its integration and performance in layered reinforcement settings, particularly for enhancing bearing capacity. By analyzing various test parameters such as SHCC layer thickness, grooving conditions, and anchoring techniques, this study provides valuable insights into the optimal use of SHCC in concrete reinforcement. It addresses both experimental and theoretical aspects of civil engineering and materials science, filling a crucial research gap and contributing significantly to the understanding of advanced cementitious composites in structural applications.

## Experimental work

### Materials

In this study, the conventional concrete mixture achieved an average cube compressive strength of 32 MPa at 28 days, formulated as shown in Table 1. The mixture comprised graded crushed basalt dolomite weighing 1270 kg/m^3^, with a maximum coarse aggregate size of 10 mm, combined with local river sand at 600 kg/m^3^ serving as the fine aggregate. The mix also included water at 175 kg/m^3^ and Portland cement of grade 42.5 N weighing 350 kg/m^3^.

Table 1 details the SHCC mix proportions, which incorporated sand, Portland cement grade 42.5 N, silica fume, silica sand (quartz), natural river sand, superplasticizer, polypropylene fibers, and water. The polypropylene fibers used were 14 mm in length and 0.01 mm in diameter. Experimental characterization tests for the SHCC matrix followed the standards set by JSCE^36^, revealing that the material had tensile and compressive strengths of 6.6 MPa and 42.2 MPa, respectively. The relatively lower tensile strength compared to typical SHCC values (8–16 MPa) is primarily attributed to the use of polypropylene fibers, which generally do not provide as high tensile strength as polyethylene (PE) fibers commonly used in SHCC. In this study, PP fibers were intentionally chosen to reduce material costs, since the targeted application of enhancing the bearing strength of concrete supports does not demand very high tensile strength levels as required in other structural elements.

**Fig. 1:**
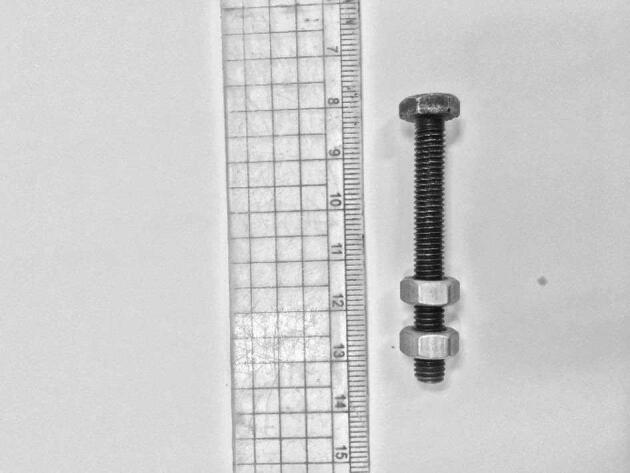
HS Gr.8.8 steel bolt (D = 10 mm, Length = 80 mm).

The study utilized high-strength friction-grip bolts of grade 8.8, as illustrated in Fig. 1. These bolts, 10 mm in diameter and 80 mm in length, were made from medium carbon steel, quenched and tempered, featuring a stainless-steel finish with a hex head. The threads extended the entire length of the bolt. According to the bolt manufacturer, the proof strength, yield strength, and ultimate tensile strength were 580 MPa, 640 MPa, and 800 MPa, respectively.

To ensure uniform distribution of the applied load, a steel plate measuring 60 × 60 mm^2^ was employed. As per the manufacturer’s technical data sheet, the plate exhibited yield and tensile resistances of 250 MPa and 360 MPa, respectively, with a Young’s modulus of 199 GPa.

**Table 1:** The mix proportions of normal concrete and SHCC (kg/m^3^).

Type	Cement	Silica fume	Dolomite	River sand	Quartz	Water	Super plasticizer	Polypropylene fiber
NC	350	-	1270	600	-	165	2	-
SHCC	800	200	-	800	230	280	25	4

### Specimens preparation and setup

**Fig. 2:**
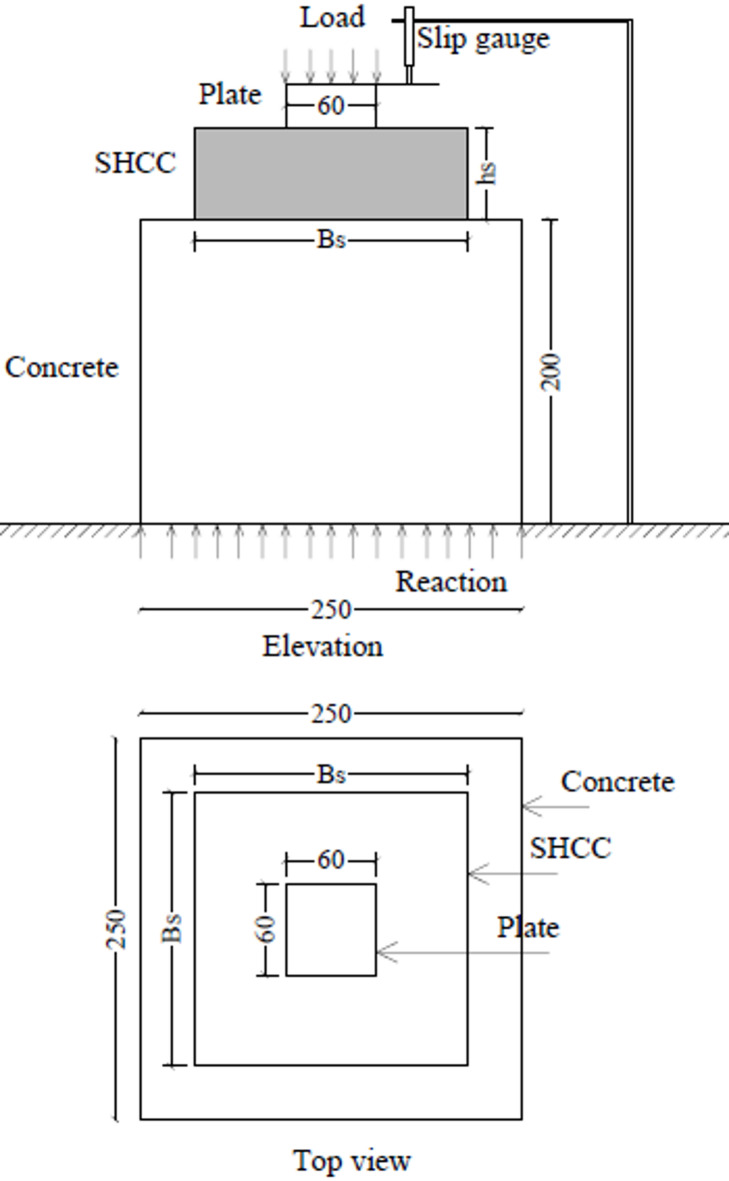
Schematic of the bearing test setup and specimen configuration. B_s_ and h_s_ denote the width and height of the SHCC block, respectively.

**Fig. 3:**
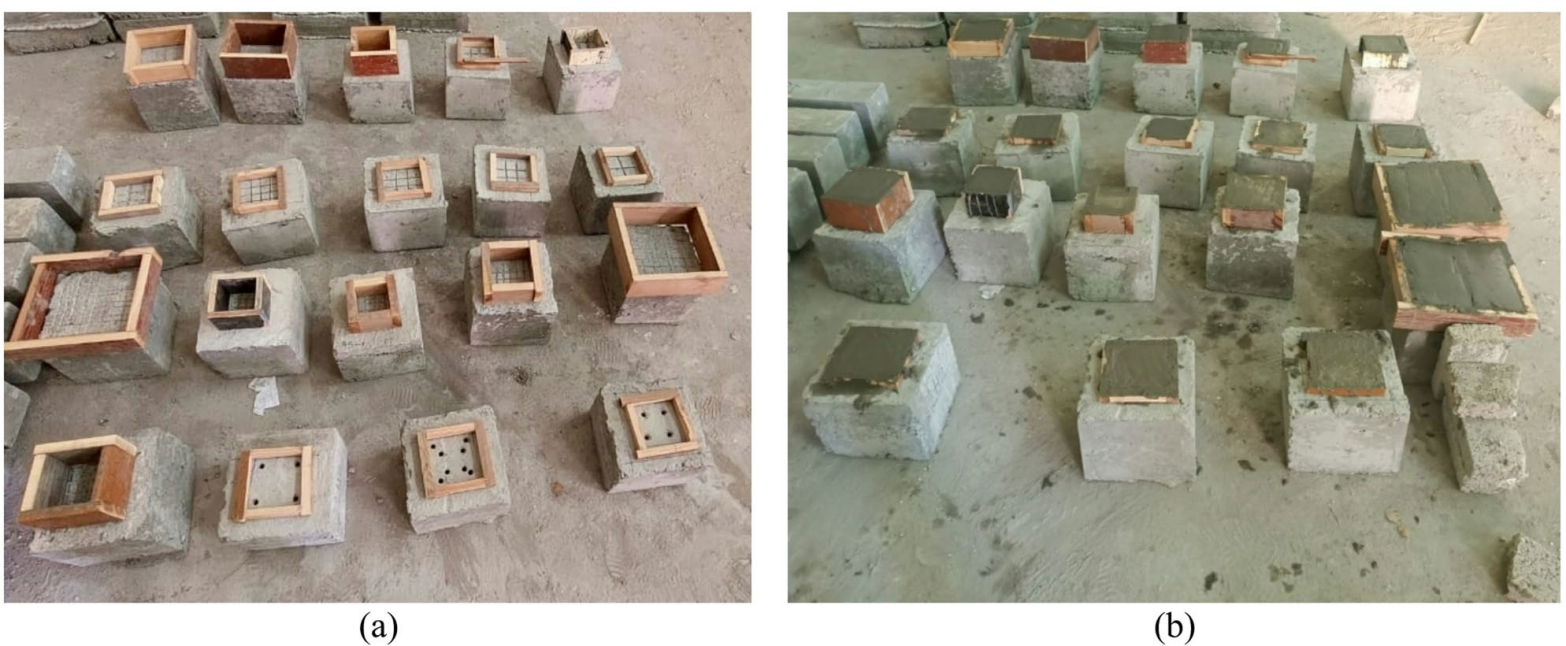
Specimens preparation; (**a**) SHCC molds and (**b**) SHCC casting.

Figure 2 illustrates the test setup utilized in this study. The concrete blocks, cast from conventional concrete mix, measured 200 mm in height and 250 mm in both width and length, presenting a square configuration in top-view. After a 28-day curing period for the concrete blocks, the top surfaces were prepared by roughening and cutting shallow grooves to enhance the mechanical interlock and bond between the concrete substrate and the SHCC overlay. Subsequently, an SHCC layer was cast on the prepared top surface, its dimensions varying according to specific test parameters. Subsequent to another 28-day curing period for the SHCC layer, laboratory testing was performed on all specimens. Both the concrete and SHCC layers were cured under laboratory conditions in Egypt, with an average temperature of approximately 25–30 °C and relative humidity of about 60–70%. Figure 3 shows the preparation of test specimens. Tests were conducted using a universal testing machine with a maximum capacity of 2000 kN. The load was applied at a rate of 1.2 kN/s via a load cell and was evenly distributed through a steel plate that measured 30 mm in thickness and 60 mm in both width and length. Displacement of the specimens was monitored using a displacement transducer capable of measuring up to 100 mm with a precision of 0.01 mm. Both the load cell and the displacement transducer were connected to a digital data acquisition system, which recorded the behavior of the test specimens under load.

### Test program

**Fig. 4:**
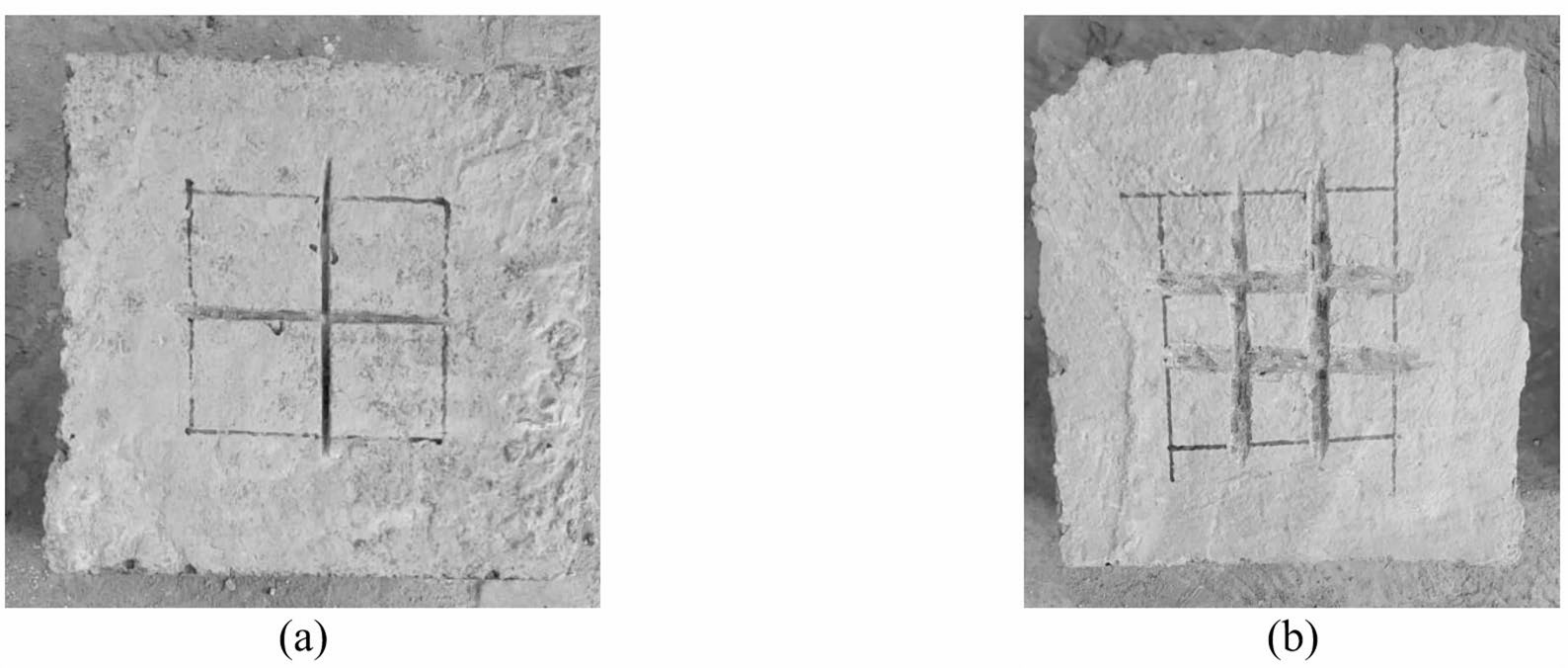
Groove configurations; (**a**) one set of orthogonal grooves and (**b**) two sets of orthogonal grooves.

**Fig. 5:**
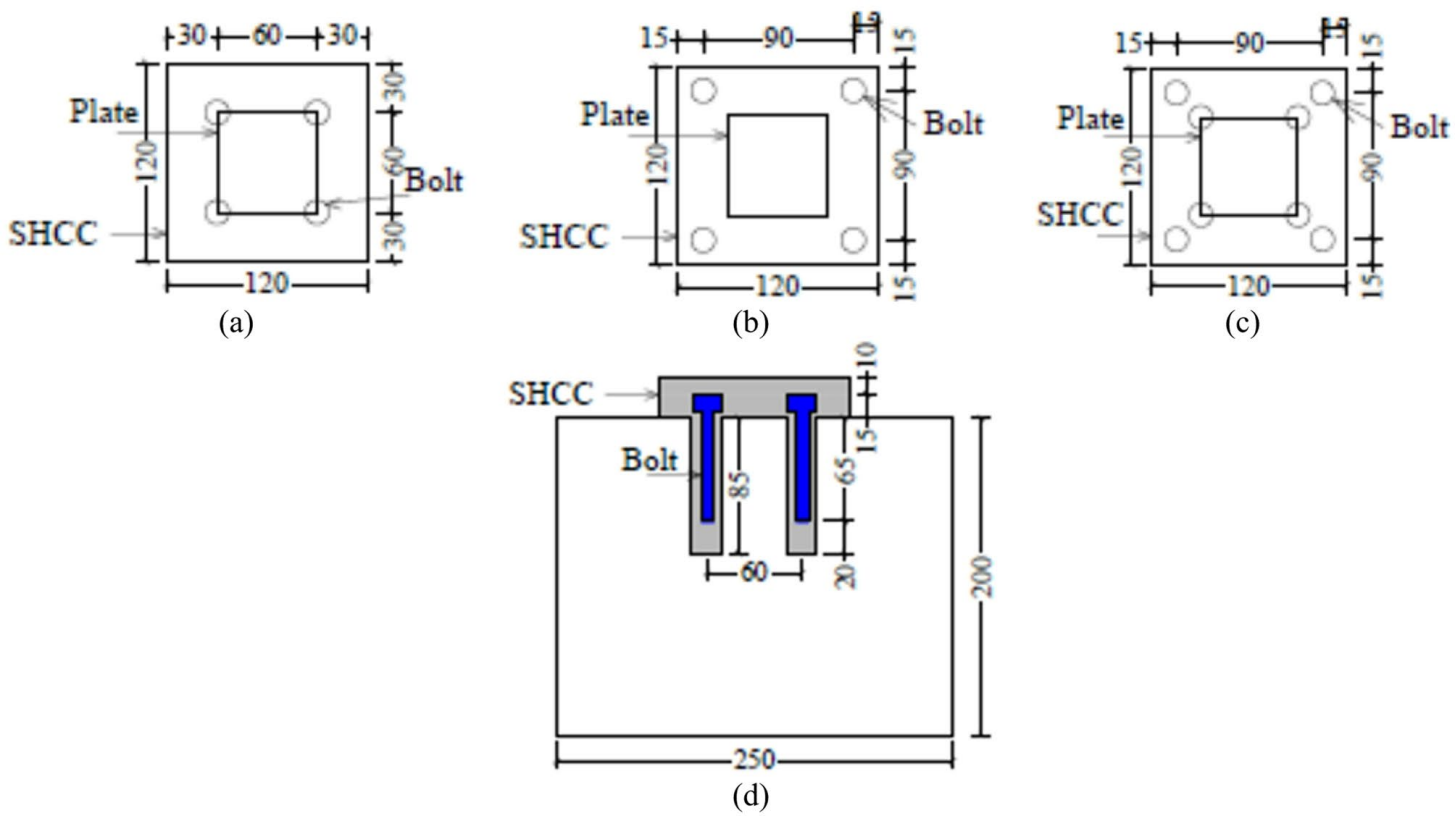
Details of group 6 specimens: (**a**) top view of Scheme I, (**b**) top view of Scheme II, (**c**) top view of Scheme III, and (**d**) elevation view of Scheme I showing bolt arrangement and embedment within the SHCC and concrete layers.

**Fig. 6:**
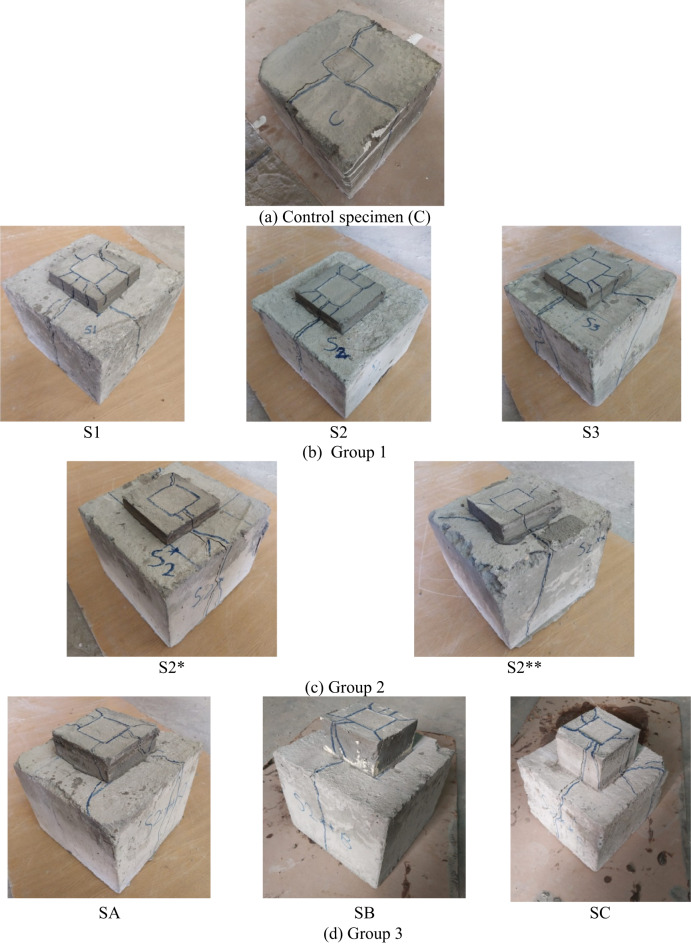

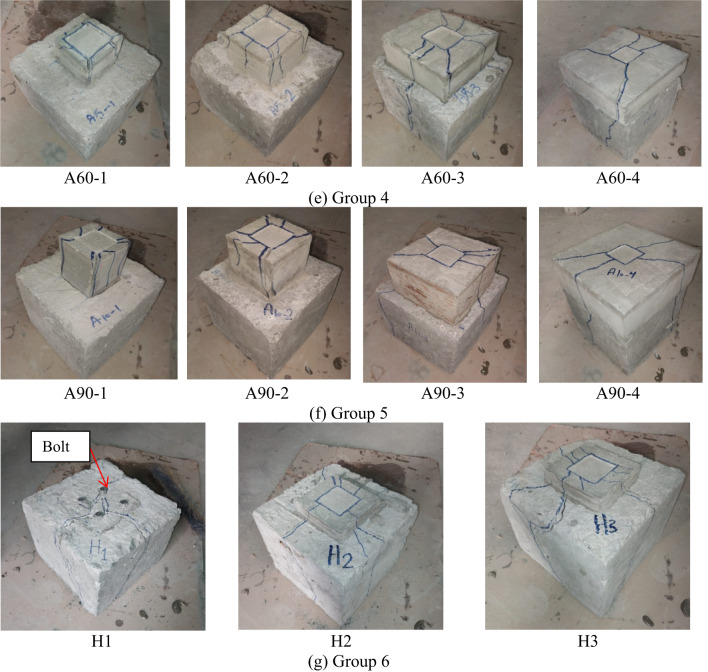
Failure mechanisms.

The specimens in this study were divided into six groups, namely from G1 to G6, as demonstrated in Table 2. Each group studied different parameter. G1 and G2 investigated the effect of grooving number and size, respectively, while G3 explored the impact of changing SHCC layer depth on the bearing behavior. Each group includes three specimens to be compared together and against the control specimen. It is worth noting that the purpose of making grooves is to increase the bond between the SHCC layer and substrate concrete block. The grooves were made equally in both directions, as shown in Fig. 4. Similarly, G4 and G5 examined the effect of SHCC layer size under two different thicknesses of 60 mm and 90 mm, respectively. They contain four specimens each. Last group, G6, studied the efficacy of using anchors on the bearing behavior. The group includes three specimens, where each has a different scheme on the plan view (I for H1, II for H2 and III for H3), as depicted in Fig. 5. For the three samples, vertical holes with diameter of 12 mm were drilled. Depth of the drilled holes was 85 mm. Bolts with 10 mm diameter were inserted in these holes. SHCC mix was used as an epoxy around the bolts (Fig. 6).

**Table 2:** Test configuration of supporting bearing strength specimens.

Group	Parameter	Sample	Grooving conditions			SHCC layer conditions		Anchors scheme
			Size wg (mm×mm)	Number Ng	Length Lg (mm)	Width Bs (mm)	Depth hs (mm)	
		C	N/A	N/A	N/A	N/A	N/A	N/A
G1	Grooving number	S1	3 × 3	1	120	120	25	N/A
		S2	3 × 3	2	120	120	25	N/A
		S3	3 × 3	3	120	120	25	N/A
G2	Grooving size	S2	3 × 3	2	120	120	25	N/A
		S2*	5 × 5	2	120	120	25	N/A
		S2**	8 × 8	2	120	120	25	N/A
G3	SHCC layer depth	SA	8 × 8	2	120	120	40	N/A
		SB	8 × 8	2	120	120	60	N/A
		SC	8 × 8	2	120	120	90	N/A
G4	SHCC layer area	A60-1	2 × 2	5	90	90	60	N/A
		A60-2	2 × 2	6	120	120	60	N/A
		A60-3	2 × 2	9	180	180	60	N/A
		A60-4	2 × 2	12	250	250	60	N/A
G5	SHCC layer area	A90-1	2 × 2	5	90	90	90	N/A
		A90-2	2 × 2	6	120	120	90	N/A
		A90-3	2 × 2	9	180	180	90	N/A
		A90-4	2 × 2	12	250	250	90	N/A
G6	Bearing anchors	H1	N/A	N/A	N/A	120	25	I
		H2	N/A	N/A	N/A	120	25	II
		H3	N/A	N/A	N/A	120	25	III

Where wg is groove cross-sectional size. Lg is groove length; N_g_ is grooves number in each direction; B_s_ is size of SHCC layer; h_s_ is thickness of SHCC layer.

## Results and analysis

### Bearing stress-slip curves

In the current study, the bearing stress was determined according to the following equation:

$$\:{f}_{b}=\:\frac{P}{{A}_{p}}$$

where *P* is the applied load and $\:{A}_{p}$ is the loaded steel plate size of 60 $\:\times\:$ 60 mm^2^.

**Fig. 7:**
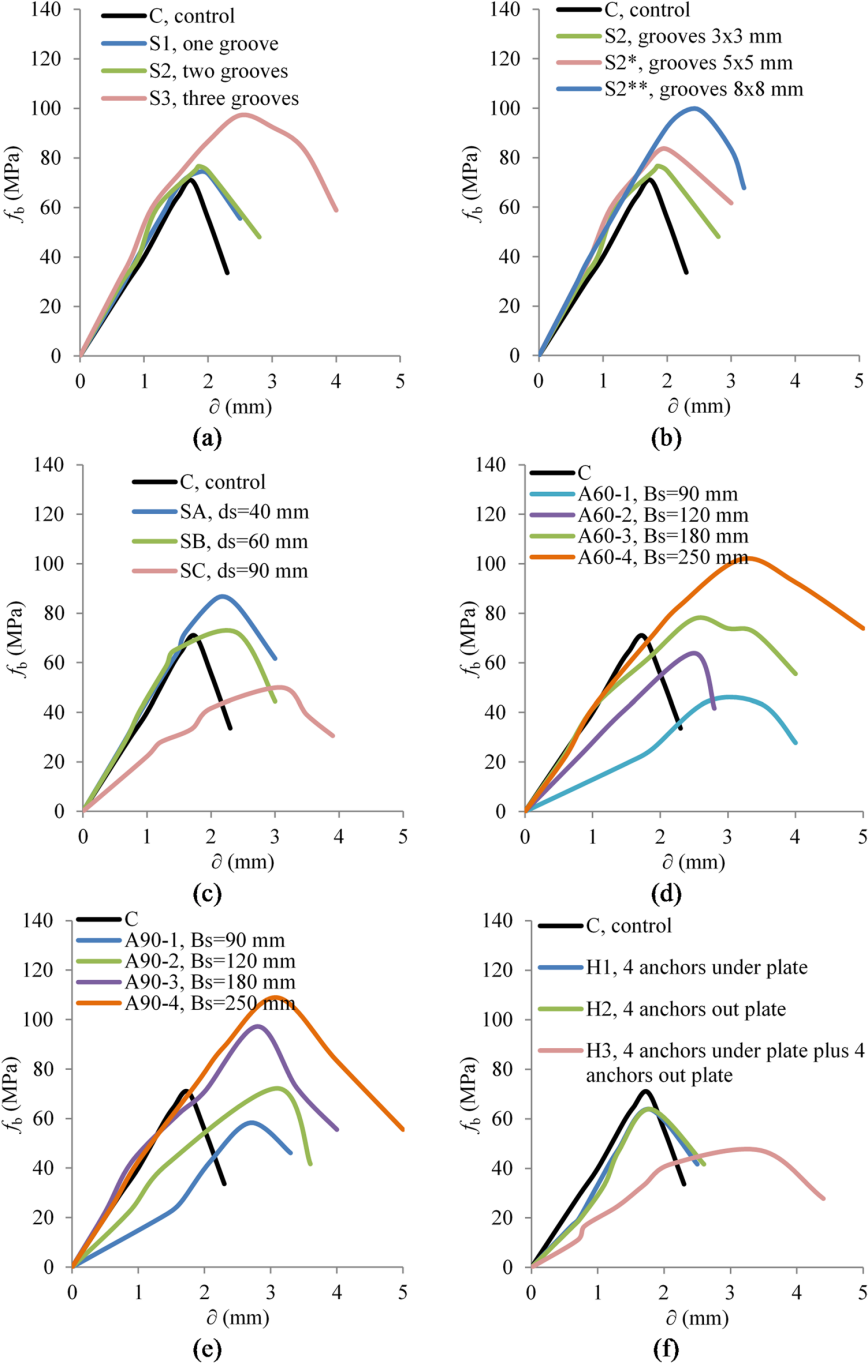
Bearing stress-slip curves of tested samples. (**a**) G1, (**b**) G2, (**c**) G3, (**d**) G4, (**e**) G5, (**f**) G6.

Figure 7 demonstrates the bearing stress-slip relationship curves. Table 3 lists the results of bearing tests. In general, the curves can be analyzed by dividing them into three regions, each highlighting critical aspects of the material behavior under load. In the elastic stage, observed at the beginning of the stress-slip curve, the relationship between stress and slip is primarily linear, indicating that the material behaved elastically. This stage reflected the inherent material properties, such as bearing stiffness and initial tensile strength, where no permanent deformation occurred. Following the purely elastic response, the curve entered the elastic-plastic stage, where the material started to exhibit plastic behavior along with elastic characteristics. This stage is marked by a gradual curvature in the stress-slip relationship, leading up to the peak stress point. It represents the transition phase where the material begins to yield under stress, exhibiting permanent deformations while still trying to resist further loading. The softening stage, or post-peak decline, begins immediately after the peak stress point and is characterized by a reduction in bearing stress as slip continues to increase. The material undergoes further plastic deformation, leading to wider crack propagation and complete fracturing.

Figure 7(a) illustrates the bearing stress-slip relationships for concrete specimens in Group 1 with varying numbers of grooves. The control specimen (C) shows a steady increase in bearing stress with slip until reaching a peak, followed by a sharp decline, indicative of the typical brittle failure of standard concrete under load. Specimen S1, with one groove, follows a similar trend but with a slightly higher peak stress. Specimen S2, featuring two grooves, demonstrates further improvement in peak stress and a more gradual decline post-peak, highlighting the effectiveness of additional grooving in distributing stress and delaying failure. The specimen with the most grooves, S3, exhibits the highest peak stress and sustains higher stress levels longer before declining. Increasing the number of grooves in concrete specimens increases the surface area for mechanical interlocking, which enhances the frictional resistance between different material layers. This increased friction helps maintain material integrity under stress by reducing slip or separation at interfaces.

**Table 3:** Test results of bearing tests.

Group	Variable	Sample	f_bu_ (MPa)	∂u (mm)	k_b_ (MPa/mm)	EAC (MPa.mm)
G1	Grooving number	C	70.8	1.75	40.1	103.4
		S1	73.9	1.82	41.1	129.4
		S2	76.7	1.85	42	145.1
		S3	97.2	2.5	48.2	281.2
G2	Grooving size	C	70.8	1.75	40.1	103.4
		S2	76.7	1.85	42	145.1
		S2*	83.6	1.92	43.5	179.1
		S2**	99.7	2.47	49.2	241.6
G3	SHCC layer depth	C	70.8	1.75	40.1	103.4
		S2A	86.2	2.1	45	177.0
		S2B	72.2	2.4	45	157.0
		S2C	50	3.1	22.3	139.0
G4	SHCC layer area	C	70.8	1.75	40.1	103.4
		A60-1	44.4	2.7	12.7	123.9
		A60-2	63.9	2.5	27.3	123.3
		A60-3	77.8	2.5	41.2	232.9
		A60-4	101.9	3.21	41.7	367
G5	SHCC layer area	C	70.8	1.75	40.1	103.4
		A90-1	58.3	2.72	15.1	118.4
		A90-2	72.2	3.1	25.6	184
		A90-3	97.2	2.8	40	254
		A90-4	108.9	3.1	40	352.2
G6	Bearing anchors	C	70.8	1.75	40.1	103.4
		H1	63.9	1.78	30.2	96.6
		H2	63.9	1.8	30.1	99.1
		H3	47.2	3.46	19.2	142.1

Figure 7(b) explores the impact of varying groove sizes on the bearing stress-slip relationships for concrete specimens in Group G2. The specimens with various grooves, S2 with 3$\:\times\:$3 mm grooves, S2* with 5$\:\times\:$5 mm grooves, and S2** with 8$\:\times\:$8 mm grooves, all exhibit enhanced performance compared to the control specimen (C). As the groove size increases from 3$\:\times\:$3 mm to 8$\:\times\:$8 mm, there is a noticeable improvement in both the peak stress achieved and the post-peak behavior of the specimens, indicating better energy absorption and distribution capabilities. This trend suggests that larger grooves provide better mechanical interlocking within the concrete matrix, which increases the frictional forces at the interfaces within the concrete, helping to hold the structure together under greater loads and more extensive deformation. This increased interlocking and friction reduce the likelihood of crack propagation that typically leads to structural failure, allowing the specimens with larger grooves to sustain higher loads longer than those with smaller grooves or the control specimens.

Figure 7(c) demonstrates the bearing stress-slip relationships for concrete specimens differentiated by the thickness of the SHCC layer while maintaining number and size of grooves. Specimen SA, with a 40 mm thick SHCC layer, showed a higher peak stress compared to the control and a more gradual decline in stress post-peak, indicating better stress management. In contrast, Specimen SB, which initially mirrored SA’s performance, exhibited a notable reduction in its slope as it neared the start of the elastic-plastic region. This sudden change could be due to the initiation of microcracking or the onset of delamination within the SHCC layer, which interrupts the stress distribution and reduces overall bearing strength. Moreover, Specimen SC, with the thickest SHCC layer of 90 mm, displayed a reduced performance from the outset. This reduction could stem from the inefficient stress distribution, where the spreading line of the stress wave might be reaching the edges of the SHCC layer prematurely. This premature stress localization at the layer edges could be exacerbating stress concentrations, leading to a less effective distribution of stresses across the composite structure and a decreased load-bearing capacity.

Figure 7(d) presents a comparison of the bearing stress-slip curves for Group 4, where the SHCC layer sizes were varied while maintaining a consistent number and size of grooves. Specimens A60-1 and A60-2, which had smaller SHCC block sizes, exhibited reduced performance in the stress-slip curves. A60-1, with the smallest block size of 90 × 90 mm², showed a significant drop in bearing capacity compared to the control specimen, reflecting rapid failure due to its inability to effectively distribute stress. This specimen behaved more like an isolated unit, failing to integrate with the underlying concrete. A60-2, featuring a slightly larger SHCC block (120 × 120 mm²), demonstrated some improvement with a modest increase in bearing capacity over A60-1, but it still failed to fully optimize stress distribution, leading to a noticeable reduction in bearing capacity compared to the control. The underperformance of these smaller blocks can be attributed to the premature reflection of stress waves at the SHCC layer edges, which hindered effective load absorption and distribution. In contrast, the stress-slip curves for A60-3 and A60-4, with larger SHCC block sizes of 180 mm and 250 mm, respectively, showed much more stable behavior. The increased dimensions of the SHCC layers in these specimens facilitated a more cohesive interaction with the underlying concrete, preventing the early drop in bearing capacity observed in smaller blocks. This allowed A60-3 and A60-4 to sustain higher peak stresses for extended periods.

A similar trend was observed in Group 5, as shown in Fig. 7(e), where the SHCC layer thickness remained constant at 90 mm, but the block sizes varied. Specimen A90-1, with the smallest block size of 90 mm, displayed a significant reduction in bearing capacity, mirroring the behavior of A60-1, due to similar stress distribution challenges. Although A90-2 exhibited slightly better performance, with a marginal increase in bearing capacity compared to the control specimen, it still failed to fully optimize stress distribution, leading to overall lower performance. However, A90-3 and A90-4, with larger SHCC block sizes of 180 mm and 250 mm, respectively, demonstrated more favorable stress-slip behavior, similar to the results seen in Group G4, indicating that larger SHCC layers contribute to better stress management and higher structural resilience. This progression across the specimens in Groups 4 and 5 underscores the crucial need to balance the SHCC layer’s thickness with the overall block size to maximize the material’s potential in enhancing concrete’s structural performance under load and ensuring that the SHCC layer and the underlying concrete block function together as a unified system.

Figure 7(f) illustrates the effects of different anchor configurations on the bearing stress-slip relationships of concrete specimens in Group 6. The graph distinctly shows that the introduction of anchors, whether beneath the plate or outside it, did not result in performance improvement; rather, it led to a reduction in bearing stress capacity across all configurations. Specimens H1 and H2 showed a reduction in peak stress compared to the control. This suggests that instead of distributing the load effectively, the anchors may act as stress concentrators, weakening the structural integrity directly beneath the load. Moreover, Specimen H3, which combines anchors both under and outside the plate, displayed the most significant reduction in performance. The simultaneous placement of anchors likely introduces multiple weak points throughout the concrete matrix, facilitating the early development of cracks and reducing the overall load-bearing capacity of the specimen.

### Failure modes

Figure 6 illustrates the diverse failure patterns observed across different experimental groups, each depicted in sub-figures labeled from (a) to (f), showing a consistent cracking trend within each group. In general, the failure mechanism was characterized by the formation of an inverted concrete cone beneath the loaded area. This cone, resembling a pyramid, was driven outward, generating radial pressure that caused the concrete block to fracture, typically splitting it into three or more radial cracks.

Specifically, Fig. 6(a) highlights the failure characteristics of the control specimen (C), where the initial cracks manifested on the block’s surface at load percentages nearing their peak capacity. This rapid onset of cracks led to immediate structural failure due to splitting along both the surface and sides of the block. Such explosive-like breakages underscore the concrete’s weakness under tensile stress; a common issue mitigated in part by the inclusion of an SHCC layer. Figure 6(b) depicts the failure modes of Group 2. The inclusion of the SHCC layer in the concrete blocks contributed to a reduction in crack width and a delay in reaching the ultimate failure point, indicative of SHCC’s enhanced ductility and crack distribution capabilities. Additionally, increasing the number of grooves improved stress distribution, promoted controlled cracking, and delayed the onset of ultimate bearing capacity. This led to a shift from sudden, brittle failure to a more gradual, ductile failure mechanism, enhancing the overall structural resilience.

Figure 6 (c), pertaining to Group G2, demonstrates a similar pattern of concrete block splitting and SHCC layer cracking, with observable sagging beneath the loaded plate. The analysis of this group indicated that varying the size of the grooves had a measurable impact on the failure mechanisms, as larger grooves provided more space for the SHCC layer to engage and distribute stresses, which effectively delayed the propagation of critical cracks and the onset of structural failure. Moreover, grooves acted as crack arresters. If a crack initiated at the interface, the groove could interrupt the crack path, effectively halting its propagation.

In analyzing the failure mechanisms of concrete specimens with varying SHCC layer thicknesses, as shown in Fig. 6(d), it was observed that the introduction of a 40 mm SHCC layer improved structural integrity by delaying crack initiation and promoting more distributed microcracking. This thickness allowed the SHCC to effectively bridge cracks and redistribute stress, leading to a more controlled failure process. However, as the SHCC layer thickness increased to 60 mm and 90 mm, the failure mechanism shifted. The thicker layers experienced premature stress concentration at the edges, likely due to inefficient stress distribution within the SHCC. This led to early delamination and more pronounced crack propagation, particularly at the interface between the SHCC and the concrete substrate. Consequently, while moderate SHCC thickness improved the failure resistance, excessive thickness compromised the material’s ability to manage stress effectively, resulting in less optimal performance and a more brittle failure mode.

In Fig. 6(e), the varying SHCC block sizes in Group 4 significantly influenced the structural response under load. Sample A60-1, with the smallest SHCC block size (90 × 90 mm²), failed rapidly by splitting, as it was unable to effectively distribute stresses, functioning more as an isolated unit rather than integrating with the underlying concrete. A60-2, with a slightly larger SHCC block (120 × 120 mm²), showed improved resilience but still operated somewhat independently, with stress distribution remaining suboptimal. As the SHCC layer size increased in Samples A60-3 and A60-4, the stress distribution improved, allowing the SHCC layer to interact more cohesively with the concrete block beneath. This led to cracks not only within the SHCC layer but also in the underlying concrete, indicating that the layers functioned more as a unified structural element, reducing localized stress concentrations and preventing isolated splitting seen in smaller SHCC sizes. A similar pattern was observed in Group G5 (refer to Fig. 6(f)), where A90-1 and A90-2 experienced splitting confined to the SHCC layer without affecting the concrete below, while A90-3 and A90-4 showed cracks extending into the concrete block. This suggests that as the SHCC layer size increases, it more effectively absorbs and redistributes stress, but also transfers more load to the concrete beneath, leading to cracking in both materials and highlighting the importance of optimizing SHCC size for cohesive structural performance.

Figure 6(g) showcases the complexities introduced by using bolts as a reinforcement strategy within Group 6. The notable splitting observed on the sides and surface of the concrete block, alongside the cracking in the SHCC layer and subsidence beneath the loaded plate, underscores the dual role of the bolts in this configuration. Although bolts are typically implemented to enhance structural cohesion and load distribution, in this instance, they inadvertently acted as stress concentrators within the concrete matrix. The presence of bolts created localized points of weakness, allowing cracks to initiate and propagate more easily through these zones. This was particularly evident in sample H3, where the increased count of eight bolts led to more pronounced cracking at the SHCC layer’s base. The subsequent exposure of underlying cracks upon the removal of the SHCC layer further confirms that the bolts compromised the overall structural integrity by disrupting the uniform distribution of stress and promoting fracture paths that accelerated the deterioration process.

### Ultimate bearing strength (fb_u_) and corresponding slip (∂_u_)

**Fig. 8:**
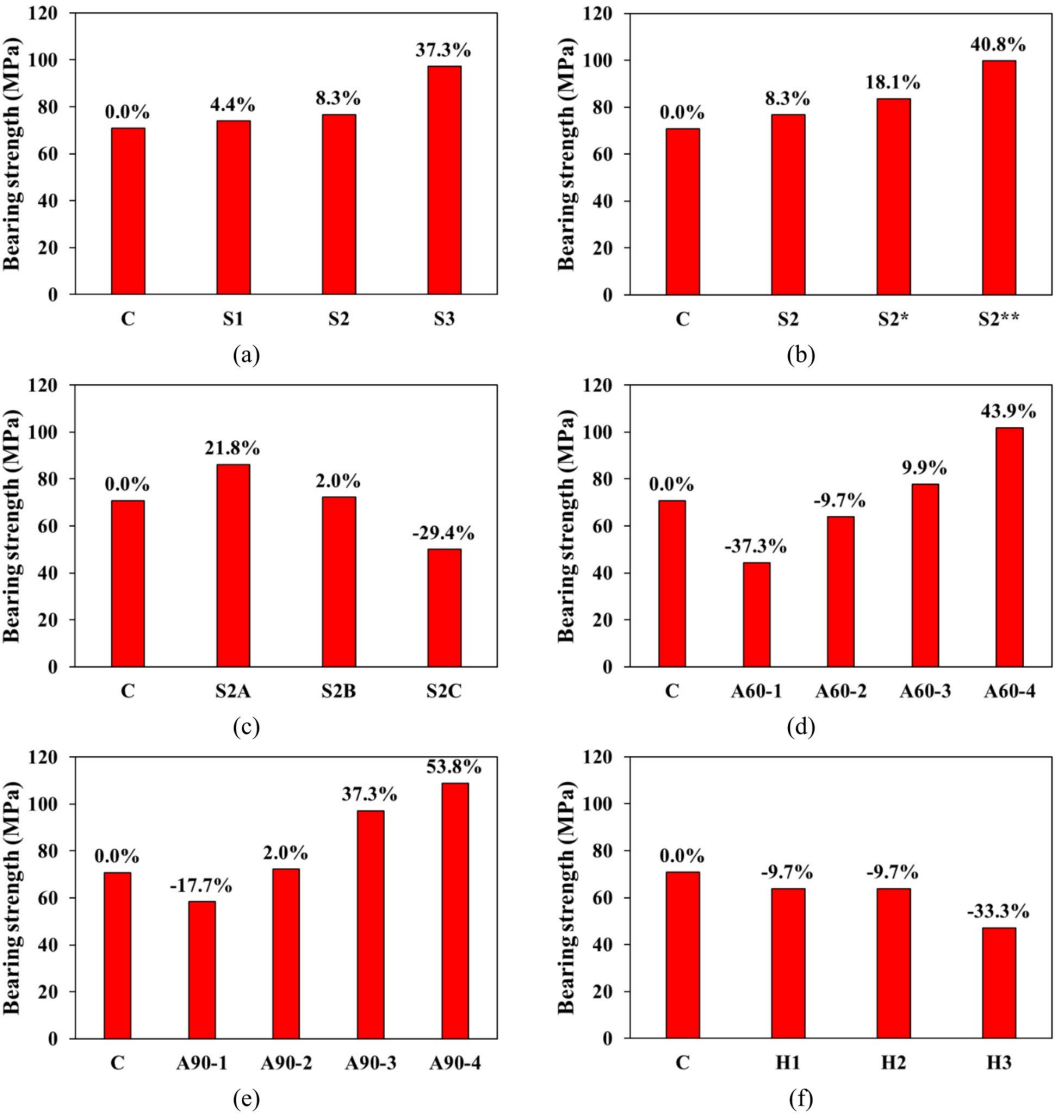
Ultimate bearing strength comparisons for each group; (**a**) G1, (**b**) G2, (**c**) G3, (**d**) G4, (**e**) G5, and (**f**) G6.

Figure 8(a–f) illustrates the comparison of ultimate bearing strength across the experimental groups, establishing the control specimen (C) as the baseline for all comparisons. In Group 1, the inclusion of grooves incrementally increases the bearing strength: a single groove (S1) enhances strength by 4.4%, two grooves (S2) by 8.3%, and three grooves (S3) significantly by 37.3%. The corresponding slip values, shown in Table 3, increase from 1.75 mm in the control to 2.5 mm in S3. Although the higher slip values accompany the strength gains, this should not be interpreted as improved bond performance. Instead, the higher slip at peak load indicates a reduction in bond stiffness and greater interfacial deformation before failure. The increase in bearing strength in this group is therefore attributed mainly to enhanced mechanical interlocking from the added grooves rather than an improvement in bond strength.

Similarly, in Group 2, increasing the groove size leads to higher bearing strength. S2 (3 × 3 mm) shows an 8.3% increase, while S2* and S2** (5 × 5 mm and 8 × 8 mm, respectively) exhibit 18.1% and 40.8% increases. Slip values also rise from 1.75 mm in the control to 2.47 mm in S2**, reflecting a larger interfacial displacement under peak load. This trend again suggests that larger grooves enhance interlocking capacity, contributing to strength improvement, even though the bond strength at the interface weakens slightly, as indicated by the higher slip.

Group 3 explores the effect of SHCC layer depth. Specimen S2A, with a moderate depth, achieves a 21.8% increase in bearing strength and a slip of 2.1 mm. In contrast, S2B shows a marginal 2.0% increase in strength, while S2C experiences a 29.4% decrease, despite a slip value peaking at 3.1 mm. The higher slip in S2C reflects bond deterioration and less effective stress transfer across the interface, suggesting that beyond an optimal depth, the SHCC layer may no longer contribute efficiently to load distribution.

In Groups 4 and 5, the influence of SHCC layer area is examined. In Group 4, increasing the SHCC block size results in mixed behavior. A60-1 and A60-2 show decreases in bearing strength of 37.3% and 9.7%, respectively, whereas A60-3 and A60-4 exhibit increases of 9.9% and 43.9%. Slip values rise from 2.7 mm in A60-1 to 3.21 mm in A60-4. The increase in strength for larger SHCC areas (A60-3 and A60-4) is attributed to improved stress distribution across a wider contact zone, while the corresponding increase in slip indicates a more flexible but weaker interfacial bond at higher deformation levels. A similar trend is seen in Group 5, where A90-3 and A90-4 demonstrate strength increases of 37.3% and 53.8%, with slip values from 2.72 to 3.1 mm. Thus, while a larger SHCC area enhances load-bearing capacity through better confinement and stress spreading, the bond becomes less rigid, as evidenced by increased slip.

Finally, Group 6, which investigates the effect of anchor configurations, shows a general reduction in performance. Bearing strength decreases by 9.7% in both H1 and H2, and by 33.3% in H3. The corresponding slip values increase markedly, with H3 reaching 3.46 mm—almost double that of the control. This increase in slip, combined with the observed reduction in strength, indicates bond weakening and inefficient stress transfer due to the presence of excessive or poorly positioned anchors. Such configurations may induce local stress concentrations that compromise interfacial integrity rather than improving it.

### Bearing stiffness (*k*_*b*_)

**Fig. 9:**
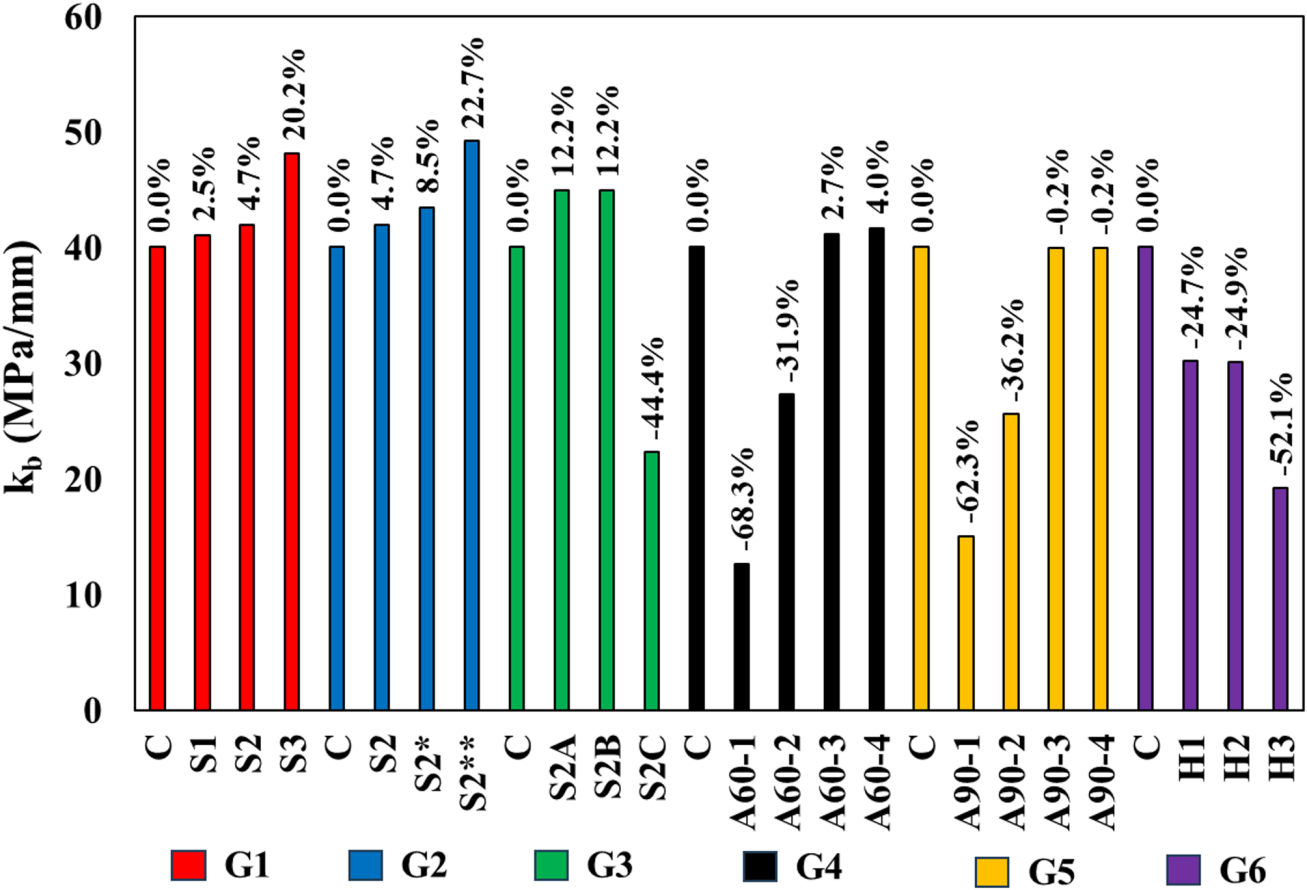
Bearing stiffness comparisons.

Bearing stiffness (*k*_*b*_​) is a measure of a material’s resistance to deformation under load, defined as the ratio of the bearing stress to the corresponding slip (deformation) in the material. It is expressed in units of MPa/mm and provides an indication of how much load a material can withstand before it begins to deform significantly. Figure 9 compares the bearing stiffness (*k*_*b*_​) of different test specimens across six groups, highlighting both improvements and reductions relative to the control specimen in each group.

In Group 1, specimens S1, S2, and S3 show incremental increases in stiffness, with S1 increasing by 2.5%, S2 by 4.7%, and S3 by 20.2%, compared to the control. This trend suggests that adding grooves enhances the stiffness of the concrete, likely due to improved stress distribution and mechanical interlocking, which increase the material’s resistance to deformation under load. The trend in Group 2 follows a similar pattern, with bearing stiffness improving as groove size increases. S2 and S2* show slight increases of 4.7% and 8.5% respectively, while S2** exhibits a significant increase of 22.7%. These results reinforce the idea that larger grooves increase the material’s load-bearing capacity and enhance its stiffness by better managing internal stresses and reducing the tendency for early deformation. The comparison in Group 3 reveals mixed outcomes based on SHCC layer depth. While S2A and S2B show a moderate increase of 12.2% in stiffness, S2C demonstrates a sharp decline of 44.4%. This reduction in S2C could be attributed to inefficient stress distribution at greater depths, where the material may become more prone to localized deformation, reducing its overall stiffness.

In Groups 4 and 5, the comparison reveals varied outcomes influenced by changes in SHCC block size. In Group 4, the smallest SHCC block size (A60-1) exhibits a significant reduction in stiffness, decreasing by 68.3%, with A60-2 also showing a notable decline of 31.9%. However, as the block size increases, the stiffness begins to recover, with A60-3 and A60-4 demonstrating slight improvements of 2.7% and 4.0%, respectively. Similarly, in Group 5, the trend largely indicates reductions in stiffness as SHCC block size increases. Specimens A90-1 and A90-3 experience considerable decreases in stiffness, by 62.3% and 36.2%, respectively. However, A90-2 and A90-4 show minimal changes, with a slight decrease of just 0.2%. These findings imply that while increasing SHCC block size beyond a certain point may diminish stiffness, likely due to over-distribution of stress or possible material instability at larger scales, there remains a critical balance in block size where the detrimental effects on stiffness can be minimized, though not entirely eliminated. Finally, Group 6 explores the effects of different anchor configurations on stiffness. All specimens in this group show a reduction in stiffness compared to the control, with H1 and H2 each decreasing by 24.7%, and H3 showing the most significant drop at 52.1%. This substantial reduction suggests that the use of anchors, particularly in higher numbers as in H3, may introduce stress concentrations that weaken the overall stiffness of the structure, making it more susceptible to deformation under load.

### Energy absorption capacity (EAC)

**Fig. 10:**
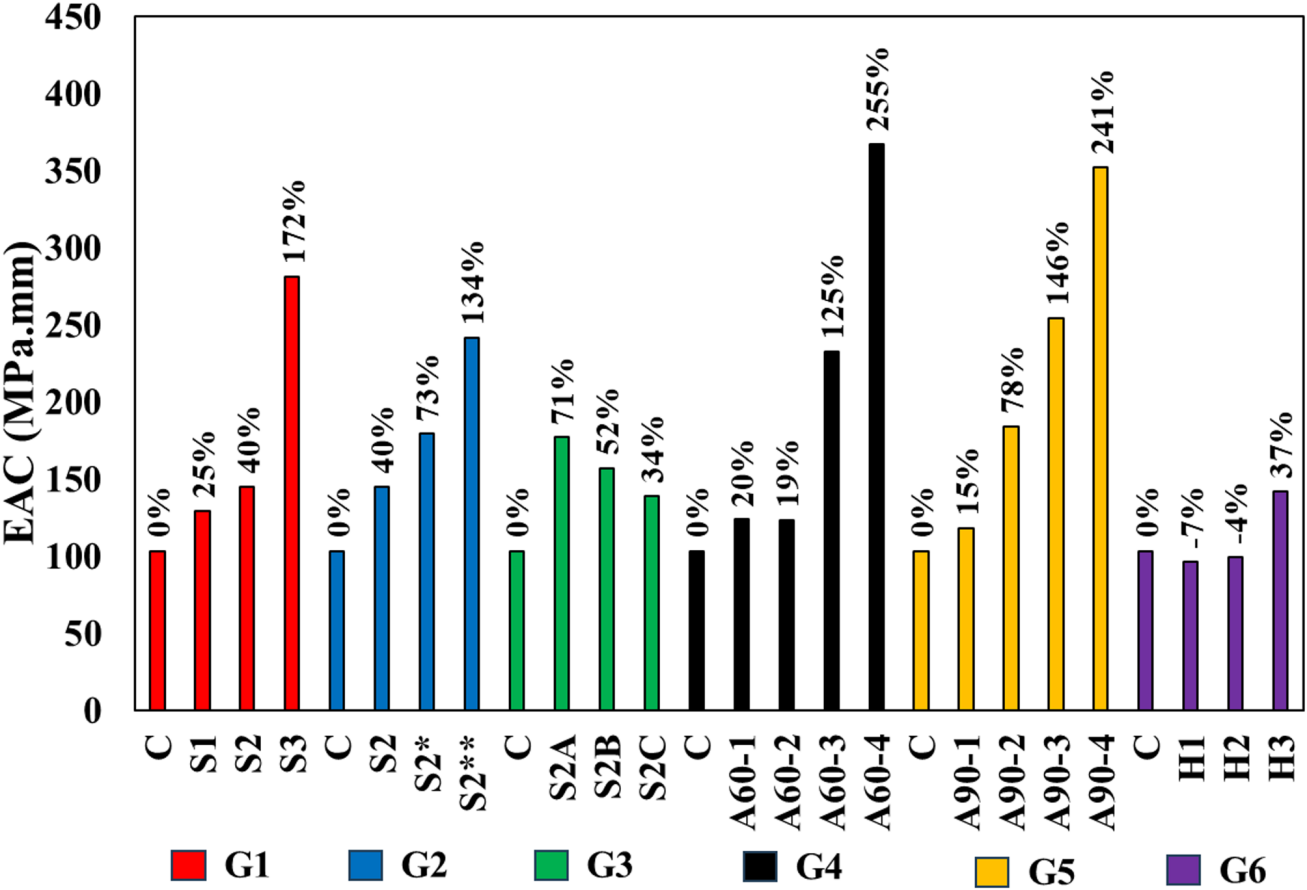
Energy absorption capacity (EAC) comparisons.

Energy Absorption Capacity (EAC) refers to the total energy a material can absorb before failure, quantified as the area under the load-displacement curve during testing^37^. It is expressed in units of MPa.mm and reflects the material’s ability to deform and dissipate energy under applied loads, indicating its toughness and ductility. Figure 10 provides a comparison of the EAC across different test specimens, revealing the impact of various structural modifications on the material’s ability to absorb energy and resist failure.

The addition of grooves significantly boosts the EAC, with S1 and S2 showing increases of 25% and 40% over the control (C), respectively. S3, with three grooves, demonstrates a remarkable 172% rise in EAC, suggesting that the presence of more grooves facilitates improved energy dissipation and crack management within the material, leading to enhanced toughness and the ability to endure more substantial deformation. An increase in groove size within Group 2 similarly enhances EAC, with S2 and S2* achieving gains of 40% and 73%, respectively, while S2** achieves a notable 134% increase. The larger grooves seem to play a crucial role in augmenting the material’s toughness, likely by enabling more controlled deformation and effective stress dissipation during loading, thereby significantly enhancing energy absorption capacity. In Group 3, the influence of SHCC layer depth on EAC reveals a range of outcomes. S2A and S2B exhibit increases of 71% and 52%, respectively, highlighting the benefits of moderate increases in SHCC depth. However, S2C, with a 34% increase, suggests that further increases in depth may yield diminishing returns, where the material’s ability to absorb energy does not increase proportionately, possibly due to less efficient stress management at greater depths, as previously pointed out.

SHCC block size plays a significant role in influencing EAC in both Group 4 and Group 5. In Group 4, A60-1 and A60-2 show modest improvements of 20% and 19%, respectively, while A60-3 and A60-4 demonstrate substantial increases of 125% and 255%. This pattern indicates that larger block sizes may contribute to better energy absorption and toughness by distributing stress more effectively across a larger area. Similarly, in G5, EAC improves notably as block size increases, with A90-2 and A90-3 showing significant gains of 78% and 146%, and A90-4 achieving a substantial 241% increase. A90-1, however, reflects smaller improvements of 15%, indicating that while larger blocks generally enhance EAC, the extent of the improvement can vary depending on the specific block size and the associated stress distribution dynamics. The introduction of bearing anchors in G6 generally leads to a reduction in EAC. Both H1 and H2 show slight decreases of 7% and 4%, respectively, with H3 experiencing a more pronounced decline of 37%. These reductions proves that the addition of anchors, particularly in greater numbers as in H3, may interfere with the material’s natural ability to dissipate energy effectively, potentially creating stress concentrations that reduce overall toughness and energy absorption.

This analysis of EAC across various groups underscores the importance of optimizing structural modifications to enhance the material’s toughness and energy absorption capacity. While certain enhancements, such as the addition of grooves and appropriate SHCC block sizes, can lead to substantial gains in EAC, other modifications, such as excessive anchoring or inappropriate SHCC layer depths, may have counterproductive effects, diminishing the material’s ability to absorb energy and resist failure.

## Proposed formula for bearing capacity

**Fig. 11:**
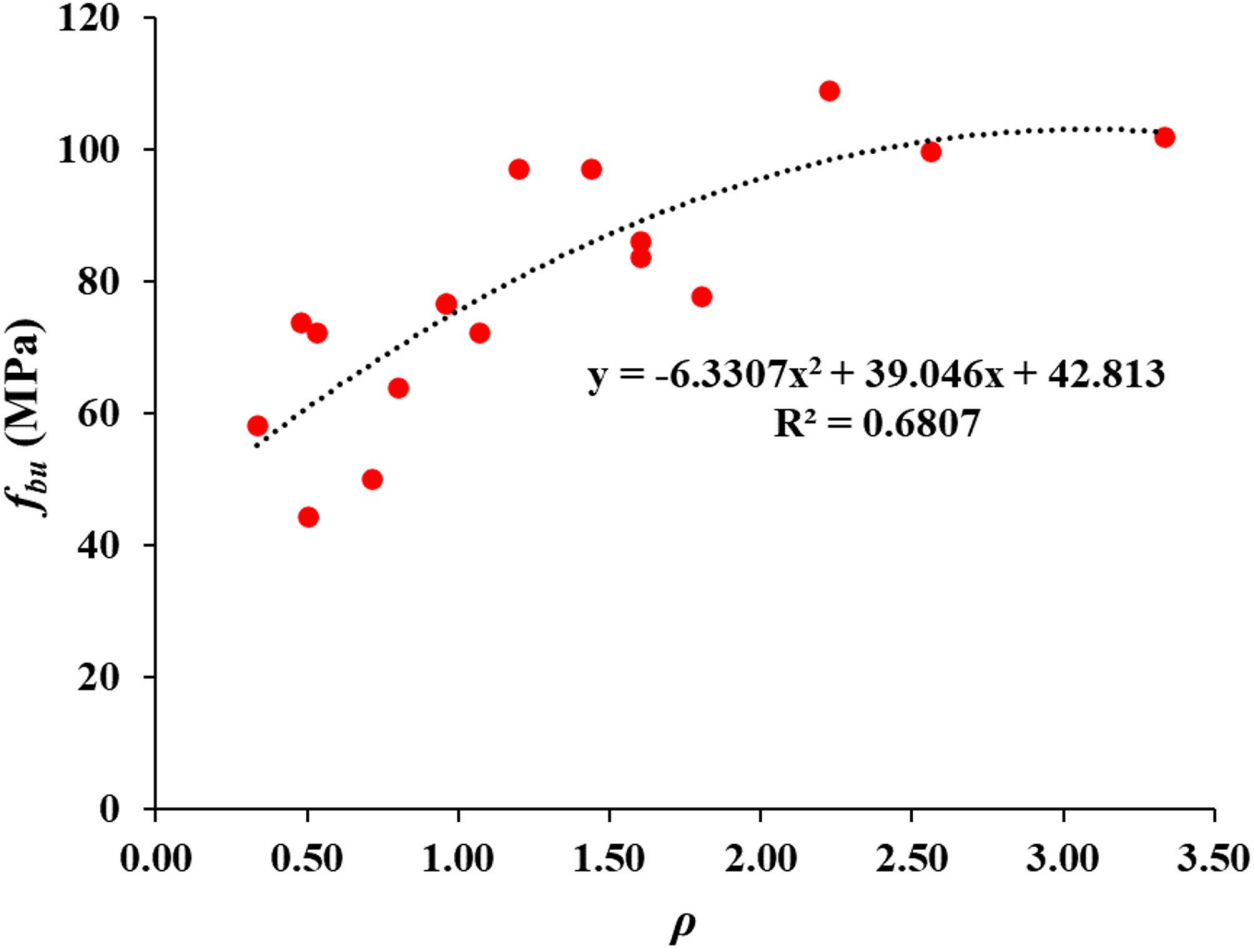
Effect of test parameters on bearing capacity.

Given that the bearing capacity formula provided in ACI 318 − 19 does not account for the specific test parameters explored in this study, such as SHCC layer thickness, groove size, number of grooves, and the ratio of SHCC area to bearing area (*A*_2_/*A*_1_), there is a need to develop a more tailored predictive model. To address this gap, the current study proposes a new empirical formula that incorporates these variables to more accurately predict the ultimate bearing capacity (*f*_*bu*_) for structures using SHCC. The relationship between the bearing capacity and the combined parameter (*ρ*), which synthesizes the effects of the aforementioned factors, is depicted in Fig. 11. The proposed equation takes the form of a quadratic polynomial as follows:

$$\:{f}_{bu}=-6.3307{\rho\:}^{2}+39.046\rho\:+42.813$$

$$\:\rho\:={2N}_{g}.\sqrt{\frac{{A}_{2}}{{A}_{1}}}.\frac{{w}_{g}}{{h}_{s}}$$

where *N*_*g*_ is the number of grooves; *A*_2_ is the SHCC area; *A*_1_ is the steel plate area; *w*_*g*_ is the size of grooves; *h*_*s*_ is the thickness of SHCC layer.

The parameter ρ was formulated to represent the combined influence of mechanical interlock (via groove number and size), confinement and crack control (via SHCC depth), and stress distribution efficiency (via the SHCC-to-bearing area ratio). Increasing groove number and size enhances bond at the interface, a thicker SHCC layer improves load transfer and crack control, while a larger A₂/A₁ ratio distributes stresses more uniformly. The quadratic polynomial was selected as it best captured the nonlinear interaction among these variables, providing the closest statistical fit to the experimental dataset.

The resulting coefficient of determination (*R*² = 0.6807) indicates a moderate level of correlation. This reflects that the model successfully captures the dominant behavioral trends observed in the tests but also highlights the need for further refinement and validation with larger datasets to improve predictive accuracy.

**Fig. 12:**
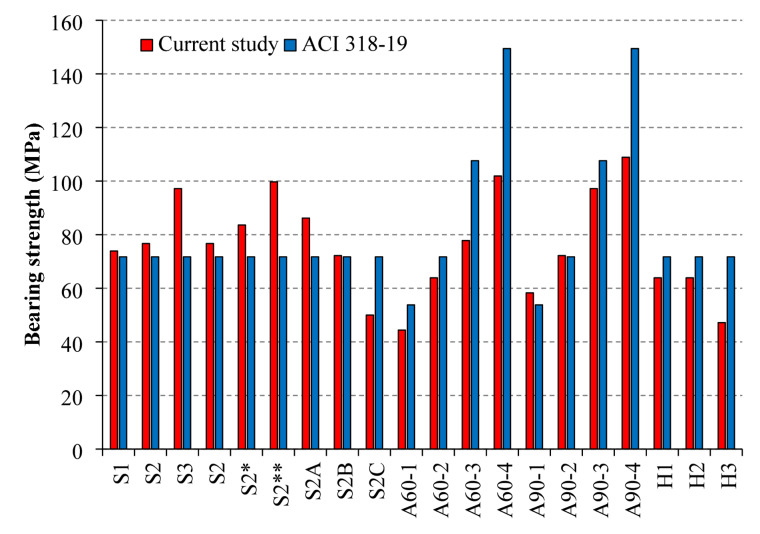
Bearing strength comparison with ACI 318 − 19 code.

Figure 12 compares the bearing strength results obtained in the current study with the predictions of ACI 318 − 19. For consistency and a valid comparison, the area of the lower base was taken equal to the SHCC layer area in both approaches. The results reveal that the ACI 318 − 19 formula consistently underestimates the bearing capacity of specimens with increased groove number and size, where enhanced interfacial bonding and stress distribution improved performance. Conversely, the code tends to overestimate the strength of specimens with thicker SHCC layers or anchoring. These discrepancies highlight the inherent limitations of the ACI approach, which does not consider SHCC-specific parameters such as layer thickness, groove geometry, or anchoring effects, and thereby emphasize the necessity of the tailored empirical model proposed in this study.

## Conclusion

This study confirms that incorporating SHCC layers in concrete supports effectively enhances bearing strength by reducing crack width and delaying the ultimate failure point. After conducting experimental tests on the specimens across six groups, the following conclusions can be drawn:

- Increasing the number of grooves significantly enhances bearing capacity. A single groove increased strength by 4.4%, two grooves by 8.3%, and three grooves resulted in a substantial 37.3% improvement compared to the control specimen. Additionally, increasing the size of the grooves yielded further gains: two grooves of 3 × 3 mm improved strength by 8.3%, larger grooves of 5 × 5 mm by 18.1%, and the largest grooves of 8 × 8 mm enhanced strength by 40.8%. These improvements are likely due to enhanced mechanical interlocking and more effective stress distribution, which reduce localized stress concentrations and delay failure.
- Properly sizing the SHCC layer relative to the concrete block is critical for effective stress distribution. The study found that increasing the SHCC block size led to an average bearing capacity increase of 36.2%, whereas excessive SHCC depth, particularly at the expense of its size, resulted in a 29.4% reduction in bearing capacity due to inefficient stress distribution.
- The use of anchors, although intended to improve the bond between the SHCC layer and the underlying concrete, acted as stress concentrators, accelerating failure and reducing bearing strength by an average of 17.6%.
- The addition of grooves and appropriately sized SHCC layers significantly improved Energy Absorption Capacity (EAC), with increases ranging from 25% to 172%, highlighting the material’s enhanced ability to dissipate energy under load. Conversely, excessive anchoring and inappropriate SHCC layer depths reduced EAC by up to 7%, emphasizing the need for balanced design to maintain optimal performance.
- An empirical formula was developed to predict the bearing capacity of SHCC-reinforced concrete structures, incorporating key variables such as groove number and size, SHCC layer depth and size, and the use of anchors.
- Further research is recommended to refine the proposed empirical model and investigate additional variables that may affect the performance of SHCC-reinforced structures. Continued studies should focus on optimizing SHCC layer configurations to maximize both bearing capacity and energy absorption while mitigating potential drawbacks like stress concentration from anchoring. In addition, future work should prioritize further validation of the proposed technique, experimental investigations under dynamic and cyclic loading, and durability studies under environmental exposures such as freeze–thaw cycles and chloride ingress.

The findings from this study, both experimental and theoretical, should be interpreted with caution and not extrapolated beyond the specific range of variables examined.

## Data Availability

The datasets used and/or analysed during the current study available from the corresponding author on reasonable request.
